# Carboxylic Acid-Functionalized Conducting-Polymer Nanotubes as Highly Sensitive Nerve-Agent Chemiresistors

**DOI:** 10.1038/srep33724

**Published:** 2016-09-21

**Authors:** Oh Seok Kwon, Chul Soon Park, Seon Joo Park, Seonmyeong Noh, Saerona Kim, Hye Jeong Kong, Joonwon Bae, Chang-Soo Lee, Hyeonseok Yoon

**Affiliations:** 1 Hazards Monitoring Bionano Research Center, Korea Research Institute of Bioscience and Biotechnology (KRIBB), Daejeon 34141, South Korea; 2 Center for Integrated Smart Sensors (CISS), KAIST, Daejeon 34141, South Korea; 3 Department of Polymer Engineering, Graduate School, Chonnam National University, Gwangju 61186, South Korea; 4 Department of Applied Chemistry, Dongduk Women’s University, Seoul 02748, South Korea; 5 Nanobiotechnology and Bioinformatics (Major), University of Science & Technology (UST), Daejeon 34141, South Korea; 6 School of Polymer Science and Engineering, Chonnam National University, Gwangju 61186, South Korea

## Abstract

Organophosphates are powerful inhibitors of acetylcholinesterase, which is critical to nerve function. Despite continuous research for detecting the highly toxic organophosphates, a new and improved methodology is still needed. Herein we demonstrate simple-to-fabricate chemiresistive gas sensors using conducting-polymer polypyrrole (PPy) nanotube transducers, which are chemically specific and capable of recognizing sub-ppb concentrations (ca. 0.5 ppb) of dimethyl methylphosphonate (DMMP), a simulant of nerve agent sarin. Interestingly, the introduction of carboxylic groups on the surface of PPy nanotube transistors resulted in enhanced sensitivity to DMMP via intermolecular hydrogen bonding. Furthermore, it was found that the sensitivity of the nanotube transducer depended on the degree of the carboxylic group introduced. Finally, a sensor array composed of 5 different transducers including the carboxylated nanotubes exhibited excellent selectivity to DMMP in 16 vapor species.

Nerve agents, such as tabun, sarin, soman, and cyclosarin, have chemical structures similar to the organophosphate pesticide malathion. The organophosphate groups can easily inactivate acetylcholinesterase, which accumulates acetylcholine at nerve terminals^1^,^2^. The accumulated acetylcholine finally paralyzes cholinergic neurotransmission throughout the human body. In addition, those nerve agents can bind directly to nicotinic and cardiac muscarinic receptors, leading to a seizure. Unfortunately, the nerve agents are colorless, odorless, and volatile. Therefore, it is impossible to recognize them with human senses. It is known that exposure to or breathing 10 mg m^−3^ of sarin vapor for 10 min (or 100 mg m^−3^ for 1 min) causes death in approximately half the exposed individuals^3^.

Many methodologies for detecting nerve agents at low concentrations, including colorimetric detection, surface acoustic wave devices, enzymatic assays, interferometry, and ratiometric chemosensors, have been developed over the last several decades^4^,^5^,^6^,^7^,^8^. Although all of these methods have individual strengths, they have also critical limitations such as slow response, lack of specificity, low sensitivity, operational complexity, high power, high cost, and lack of portability. Therefore, there is a great demand for alternative sensing technologies in terms of public safety and environmental monitoring. Recently, one of the leading candidates was an electrically chemiresistive sensor geometry consisting of a simple chemiresistor, which relies on direct chemical interaction between the resistor and target analyte^9^. Different kinds of chemiresistors can be integrated into an array to achieve enhanced target selectivity, and can be further miniaturized to achieve low power requirements. Several materials have been utilized as chemiresistors, including metal oxides, organic semiconductors, and carbon nanotubes (CNTs)^10^,^11^. In general, metal oxides can serve as signal transducers only at high temperatures (typically over 200 °C) because of their high activation energy, and nanocarbon species must undergo sophisticated chemical treatments for sensor applications.

Conducting-polymer (CP) nanomaterials consisting of polyaniline, polypyrrole (PPy), and poly(3,4-ethylenedioxythiophene) (PEDOT) have also attracted much attention as chemiresistors^12^,^13^,^14^,^15^,^16^. Owing to their enlarged effective surface areas, nanostructured CPs can interact with lower-concentration target analytes to afford a measurable change in conductivity. In particular, one-dimensional CP nanomaterials such as nanofibers and nanotubes enable efficient charge transport along the long-axis direction, which contributes to achieving enhanced sensitivity in chemiresistive sensor applications^17^,^18^,^19^,^20^. Versatile chemical functional groups can be readily introduced to CPs during polymerization or by post-polymerization treatment, leading to better sensing performance^21^,^22^,^23^. In this work, we report on simple and efficient chemiresistive gas sensors using carboxylated PPy nanotube (cPNT) transducers capable of recognizing the sarin simulant dimethyl methylphosphonate (DMMP) at sub-ppb concentrations. Specifically, cPNTs were prepared by anionic surfactant templating, in which the carboxyl groups contributed to the increased specificity of the nanotubes toward DMMP. The sensing performance of the nanotube transducers depended on the diameter of the nanotubes and the degree of the functional group introduced. Lastly, an array of five different kinds of chemiresistors was fabricated to demonstrate the selective detection of DMMP among many interference vapors.

## Results and Discussion

**Figure 1:**
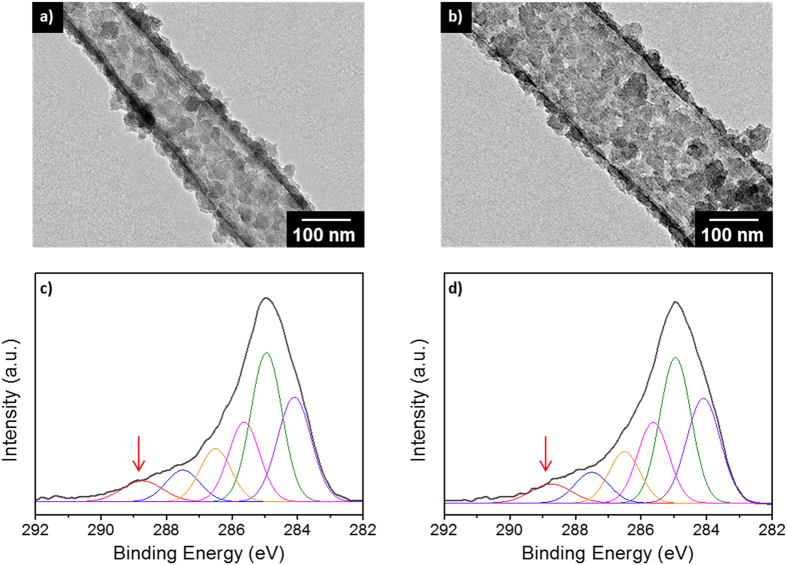
TEM images and XPS C1s spectra of (**a**,**c**) cPNT1s and (**b**,**d**) cPNT2s with different diameters, where the molar ratio of P3CA-to-pyrrole was 1/30. The arrow indicates the C1s component that originates from carboxyl groups.

One-dimensional CP nanomaterials have been prepared in sodium bis(2-ethylhexyl)sulfosuccinate (AOT) reverse emulsion systems, in which functional groups have been successfully introduced to the CPs via co-polymerization^21^. In sensor applications, especially, the functionalization of pure CPs is highly important for achieving chemical specificity toward target species^22^. To obtain enhanced sensitivity, it is also important to increase the effective surface area of CPs, depending on their size and morphology. Figure 1 shows transmission electron microscopy (TEM) images of cPNTs with different diameters. Carboxyl groups were introduced to PPy via the copolymerization of pure pyrrole and carboxylic-acid-containing pyrrole (pyrrole-3-carboxylic acid, P3CA), in which the degree of carboxyl group introduced was controlled by changing the molar ratio of pyrrole-to-P3CA in the polymerization. Two cPNTs with different diameters were fabricated to examine the size effect of the nanotube transducer on sensing performance. The diameters of the nanotubes were 112 ± 25 nm (cPNT1s) and 205 ± 26 nm (cPNT2s), respectively. Interestingly, as seen in Fig. 1, the surface roughness of the nanotubes is considerably high, which is expected to yield an enhanced effective surface area.

To examine the functionalization of cPNTs, X-ray photoelectron spectroscopy (XPS) analysis was conducted, where the XPS C1s main peak (C_total_) was deconvoluted into six individual components (from C_1_ to C_6_). The assignment of XPS C1s components is as follows: C_1_ (288.7 eV: O−C=O), C_2_ (287.6 eV: C=N^+^), C_3_ (286.5 eV: C−N^+^ and C=N), and C_4_ to C_6_ (285.6, 284.9, 284.0: C_α_ and C_β_ in a pyrrole ring)^21^. Based on those assignments, the degree of carboxyl group introduced to PPy was estimated for both cPNTs by calculating the integrated peak area ratio of C_1_ to C_total_. The area ratios of cPNT1s and cPNT2s were 0.060 and 0.056, respectively, confirming the carboxylation of PPy in the nanotube.

**Figure 2:**
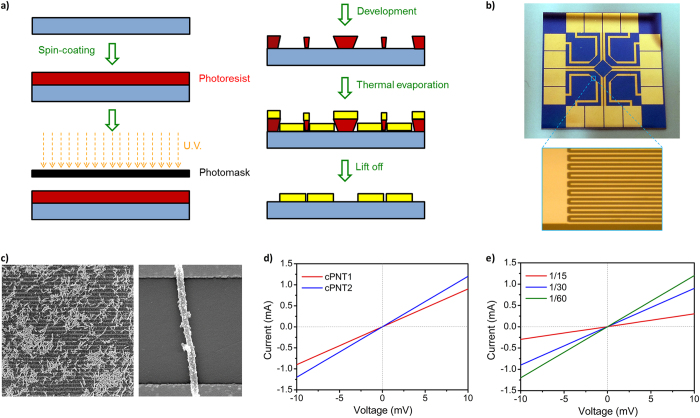
cPNT IME sensor substrate. (**a**) Schematic illustration of the fabrication process of a microelectrode sensor substrate through lithographic process. (**b**) Photo of the chemiresistive DMMP gas sensing substrate, and magnified optical image with finger parts of IMEs. (**c**) Representative SEM images of cPNTs deposited on microelectrode substrate: (left) at low magnification and (right) at high magnification (microelectrode gap: 2 μm). *I*−*V* curves of cPNTs recorded on microelectrode: (**d**) cPNTs prepared at P3CA/pyrrole molar ratio of 1/30, and (**e**) cPNT1s prepared at different P3CA/pyrrole molar ratios.

To demonstrate chemiresistive DMMP gas sensors using the cPNTs, an array of interdigitate microelectrodes (IMEs) was fabricated as a sensor substrate through a lithographic process (Fig. 2a). An IME consisted of a pair of two gold electrode bands with 100 fingers on a SiO_2_ substrate, where each finger has a 55-nm thickness (Au/Cr 50 nm/5 nm) with a 2-μm width and 3.5-mm length (Fig. 2b). The glass substrate can be readily surface-modified using a silane coupling reagent to immobilize cPNTs as the resistive transducer. To construct a chemiresistive sensor platform, aminosilane ((3-aminopropyl)trimethoxysilane, APTS) was treated on the microelectrode substrate, and then cPNTs were covalently anchored to the surface amine group of the microelectrode substrate^17^,^21^. Figure 2c displays representative scanning electron microscopy (SEM) images of the cPNTs-anchored microelectrode substrate. The microelectrodes were clearly bridged by cPNTs, which serve as chemiresistors. The IME geometry enabled cPNTs to make effective contact with the electrode. Mostly random nanotube networks, or sometimes single nanotubes, were found to readily connect the microelectrodes. The sensor substrate consisting of numerous nanotubes on IMEs has more opportunities to interact with target analytes, and thus might provide measurable signals even at extremely low concentrations.

Current−voltage (*I*−*V*) curves of the cPNT-anchored IMEs were recorded to evaluate how the nanotubes made electrical contact on the microelectrode substrate. As seen in Fig. 2d, the current increased linearly with the voltage, indicating that the cPNTs made stable ohmic contacts on the microelectrode substrate. The slope d*I*/d*V* value, indicative of the conductivity, was almost similar for both cPNT1 (0.09) and cPNT2 (0.12). Figure 2e shows the effect of the carboxyl group on the conductivity. The d*I*/d*V* value was inversely proportional to the amount of carboxyl group introduced. Since the carboxyl group prevents effective π-electron conjugation over the polymer chain, it can lead to a decrease in the electrical conductivity of the nanotubes. The carboxyl group introduced definitely affects the chemical as well as the electrical properties of PPy, which offers possibilities for tuning the sensing performance of the nanotubes.

**Figure 3:**
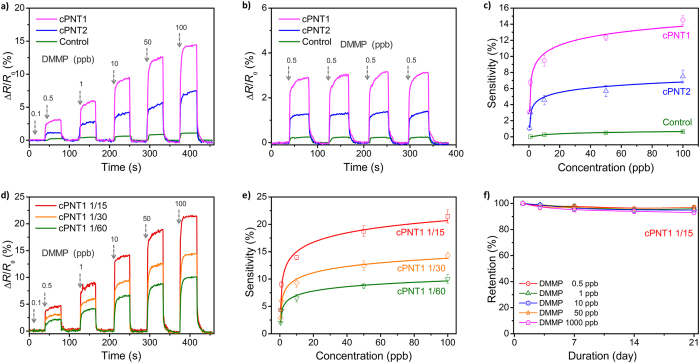
Chemiresistive sensing performance. (**a**,**b**) Real-time responses of cPNTs with different diameters upon periodic exposure to DMMP: (**a**) 0.1 to 100 ppb and (**b**) 0.5 ppb. (**c**) Changes in sensitivity as a function of DMMP concentration calculated from the real-time responses. PPy nanotubes with no carboxyl groups were employed as a control. (**d**) Real-time responses of cPNT1s with different functionalization degrees of carboxyl group. (**e**) Changes in sensitivity as a function of DMMP concentration calculated from the real-time responses. (**f**) Long-term stability of cPNT1’ responses.

The ability of cPNTs to electrically detect DMMP was first examined on the microelectrode substrate. The real-time change in resistance was recorded upon cyclic exposure to DMMP and a nitrogen stream, as exhibited in Fig. 3a,b. When cPNTs were exposed to DMMP, an abrupt rise in resistance was observed, which was proportional to the concentration of DMMP (Fig. 3a). The response was also fairly reproducible. Representatively, Fig. 3b accurately presents the reproducible electrical response of cPNTs when exposed periodically to a constant concentration (5 ppb) of DMMP. The nanotube networks on IMEs can make the response averaged over a large number of the nanotubes, which would contribute to a good reproducibility of the response. Calibration curves calculated from real-time sensing data are plotted in Fig. 3c. While the calibration curve was linear at highly low concentrations of 0.5 to 10 ppb, it was nonlinear at the entire given concentration range (0.5 to 100 ppb). It is considered that the response of cPNTs became saturated at concentrations of more than 25 ppb. The sensitivity can be defined as the normalized resistance change recorded when the saturated value was reached after exposure to DMMP. Compared with cPNT2s, cPNT1s showed higher sensitivities, which would be a result of the different effective surface areas stemming from different diameters. In other words, the same amount (weight) of cPNTs was deposited on the IME and thus cPNT1s of smaller diameter would have higher specific surface area that can interact with analytes. Figure 3d displays the responses of cPNT1s with different functionalization degrees of carboxyl group, and calibration curves obtained therefrom are plotted in Fig. 3e. The sensing performance of cPNTs clearly depended on the amount of carboxyl group introduced. Namely, the sensitivity increased as the amount of carboxyl group increased. In addition, as a control, PPy nanotubes with no carboxyl groups showed measurable resistance changes only at high concentrations of more than 10 ppm. These results indicate that the carboxyl group played a pivotal role in producing chemiresistive responses toward DMMP. Lastly, the long-term stability of cPNTs’ responses was examined. Representatively, the sensitivity of cPNT1s prepared at a P3CA/pyrrole molar ratio of 1/15 was monitored over three weeks, as shown in Fig. 3f. The cPNTs exhibited more than 95% retention of the initial sensitivity during the monitoring period.

**Figure 4:**
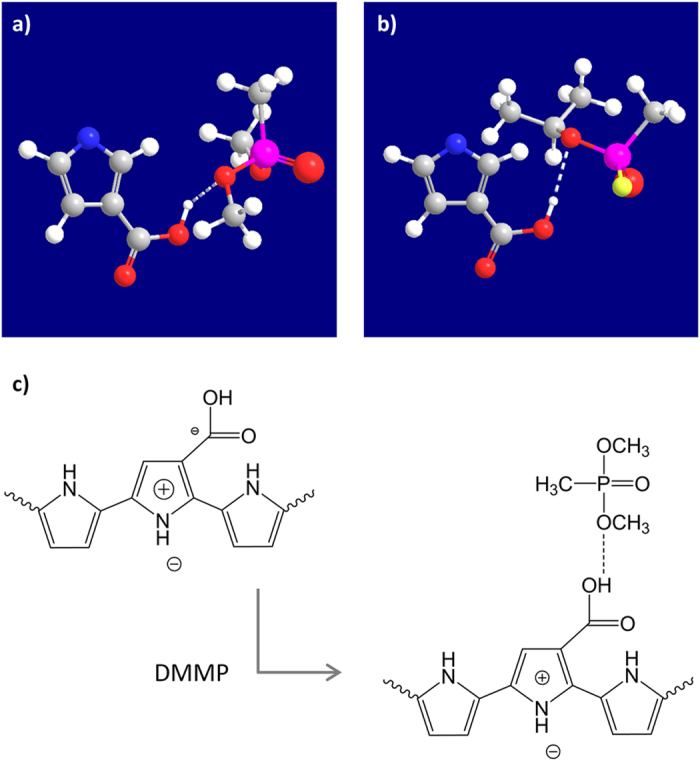
Sensing mechanism. Computed 3D graphics showing the interaction of P3CA with (**a**) DMMP and (**b**) sarin, where the white dotted line indicates a hydrogen bond between the corresponding atoms. (**c**) Scheme of describing the effect of hydrogen bonding on the oxidation state of PPy.

The carboxyl group of cPPy can noncovalently interact with DMMP^22^. Representatively, the phosphoryl group of DMMP can act as a weak hydrogen-bond acceptor, which can result in hydrogen bonding with the carboxyl group of cPPy. The intermolecular interaction between cPPy and DMMP was estimated using the MMFF94 force field calculation method. Figure 4a exhibits three-dimensional graphics showing the formation of a hydrogen bond between P3CA and DMMP (and sarin). It was found that the hydrogen atom of the carboxyl group of cPPy can be noncovalently coupled with the oxygen atom in the phosphoryl group of DMMP through a hydrogen bond, which was similar to the case of sarin (Fig. 4b). The hydrogen bonding changes the charge distribution in PPy, which affects the transport of charge carriers along the PPy backbone, as illustrated in Fig. 4c. The electron-donating effect of the carboxyl group contributes to the stabilization of the positive charge over the polymer chain. The electronegative carboxyl group can further create a kind of doping effect in PPy. However, the hydrogen bonding diminishes the effects of the carboxyl group, which may retard the effective movement of the charge carrier holes along the polymer chain. It is considered that the retarded charge transport finally results in increased resistance.

**Figure 5:**
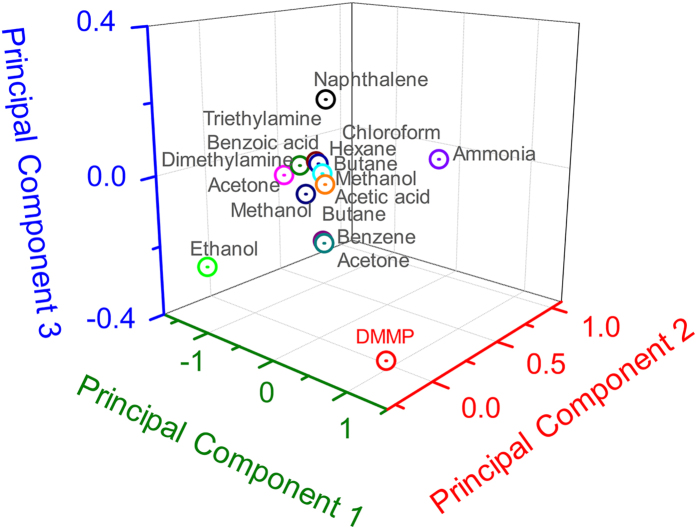
Principal components analysis plots using sensitivity inputs from 5 different chemiresistive transducers (PEDOT nanoellipsoids, PEDOT nanotubes, PPy nanospheres, PPy nanotubes, cPNT1s) to 16 analytes (including DMMP). Each analyte concentration was fixed at 1 ppm.

For the practical application of conducting polymer-based sensors, a key factor is achieving selectivity toward a target analyte. In the best sample, cPNT1s were integrated into a sensor array as one of five transducers. The ability of the sensor array to detect DMMP was tested against 15 interferences, where the response of cPNT1s was complemented by responses from other transducers. Principal component analysis was performed on the detection data set that was collected from the five different chemiresistive transducers: PEDOT nanoellipsoids, PEDOT nanotubes, PPy nanospheres, PPy nanotubes, and cPNT1s ([P3CA]/[pyrrole]: 1/15)^16^,^23^. The maximum resistance change, namely the sensitivity, was considered as input variables for the PCA. The first three principal component scores, accounting for approximately 98% of the total data variance, are plotted in Fig. 5. It was possible to categorize the analytes into several separate regions of the plot, depending on the type of analyte. In particular, DMMP had clearly discriminable components, demonstrating the selective recognition ability of the sensor array.

## Conclusions

cPNTs were fabricated, and their chemiresistive ability to recognize DMMP was successfully demonstrated. It was found that the interaction of the carboxyl group with DMMP can modulate the conductivity of PPy. On the IME consisting of a pair of two electrode bands with 100 fingers, the detection limit was as extremely low as 0.5 ppb. It is almost impossible to obtain a highly selective chemiresistive sensor using only one transducer material. The outstanding ability of cPNTs to recognize DMMP was combined with the response of other transducers in an array configuration, which allowed for the selective detection of DMMP among many interferences.

## Materials and Methods

### Materials

Pyrrole (98%), AOT (98%), and hexane (99%) were purchased from Aldrich. P3CA (95%) was obtained from Acros Organics.

### 4-(4,6-Dimethoxy-1,3,5-triazin-2-yl)-4-methyl-morpholinium chloride (DMTMM)

DMTMM was synthesized according to the previously described procedures^21^,^24^ and employed as a condensing agent. Further detailed information is given in Supplementary Information.

### Fabrication of cPNTs

cPNTs with two different diameters (cPNT1s, *ca*. 100 nm; cPNT2s, *ca*. 200 nm) were fabricated through the oxidative copolymerization of pyrrole with P3CA in an AOT water-in-oil emulsion system. First, 15.8 mmol of AOT was dissolved in 40 mL hexane with 2 mL of 7 M aqueous FeCl_3_ solution. Subsequently, 0.25 mmol of P3CA was homogeneously mixed with 7.5 mmol of pyrrole, and the P3CA/pyrrole mixture was added into the AOT/hexane solution. The oxidative copolymerization of pyrrole/P3CA proceeded at different temperatures over different time periods. The resulting products were thoroughly washed with excess ethanol to remove residual reagents. To obtain cPNT1s with different amounts of carboxyl groups, 0.25 mmol of P3CA was dissolved in 3.75, 7.5, and 15 mmol of pyrrole at P3CA/pyrrole molar ratios of 1/15, 1/30, and 1/60.

### Chemiresistive sensing

The resistance change was recorded in real time at a constant applied current of 10^−1^ μA, which was given as ∆*R*/*R*_0_ = (*R* − *R*_0_)/*R*_0_, where *R* and *R*_0_ are the real-time resistance and the initial resistance, respectively. After exposing cPNTs to DMMP (0.5 to 100 ppb) of a nitrogen stream, they were exposed to a pure nitrogen stream to remove the DMMP molecules bonded to the carboxylated PPy. The stream was supplied at a flow rate of 3 L min^−1^.

### Characterization

TEM images were obtained with a JEOL JEM-200CX. The nanoparticles diluted in ethanol were cast onto a copper grid for TEM observation. A JEOL 6700 was used to obtain SEM images. The measurement of the electrical conductivity was conducted with a Keithley 2636A sourcemeter using a four-probe method. It was found that the pellet conductivities of the cPNTs by the four-probe method ranged from 10^−1^–10^0^ S cm^−1^. XPS analysis was carried out using a Kratos AXIS-HSI spectrometer with a Mg/Al X-ray radiation source.

### An array of interdigitated microelectrodes (IMEs)

An IME consisting of a pair of gold interdigitated microelectrode bands with 25 fingers each was patterned on a SiO_2_ substrate via a photolithographic process. The dimensions of the IME were 2 μm (wide) × 3.5 mm (length) × 55 nm (thickness: Au 50 nm/Cr 5 nm). The IME substrate was treated with 10 wt% aqueous APTS solution to introduce amino groups (−NH_2_) for 12 h, and then 10 μL of 1 wt% aqueous cPNT solution was dropped on the IME.

## Additional Information

**How to cite this article**: Kwon, O. S. *et al*. Carboxylic Acid-Functionalized Conducting-Polymer Nanotubes as Highly Sensitive Nerve-Agent Chemiresistors. *Sci. Rep.* **6**, 33724; doi: 10.1038/srep33724 (2016).

## Supplementary Material

Supplementary Information
