# Supplementary material for: Carboxylic Acid-Functionalized Conducting-Polymer Nanotubes as Highly Sensitive Nerve-Agent Chemiresistors
Source: Sci Rep. 2016 Sep 21;6:33724. doi: 10.1038/srep33724 (PMC5030647; doi:10.1038/srep33724)
Supplement: Supplementary Information [file srep33724-s1.pdf]

Supplementary Information

Carboxylic Acid-Functionalized Conducting-Polymer Nanotubes as Highly Sensitive Nerve-Agent Chemiresistors

Oh Seok Kwon,[1,§] Chul Soon Park,[1,§] Seon Joo Park,[1,2] Seonmyeong Noh,[3] Saerona Kim,[3] Hye Jeong Kong,[3] Joonwon Bae,[4] Chang-Soo Lee,[1,5,*] Hyeonseok Yoon,[3,6,*]

[1]Hazards Monitoring Bionano Research Center, Korea Research Institute of Bioscience and Biotechnology (KRIBB), Daejeon 34141, South Korea

[2]Center for Integrated Smart Sensors, Daejeon 34141, South Korea

[3]Department of Polymer Engineering, Graduate School, Chonnam National University, Gwangju 61186, South Korea

[4]Department of Applied Chemistry, Dongduk Women's University, Seoul 02748, South Korea

[5]Nanobiotechnology and Bioinformatics (Major), University of Science & Technology (UST), Daejeon 34141, South Korea

[6]School of Polymer Science and Engineering, Chonnam National University, Gwangju 61186, South Korea

[§]These authors contributed equally to this work

Corresponding Authors:

*E-mail: cslee@kribb.re.kr, hyoon@chonnam.ac.kr

Synthesis of DMTMM

DMTMM provides excellent reaction yields in water as well as alcohol phases for the condensation reaction between carboxylic acid and amine groups. First, 0.75 g of 2-chloro-4,6-dimethoxy-1,3,5-triazine was reacted with 1 g of *N*-methylmorpholine in 20 mL of tetrahydrofuran (THF) for 30 min. The resulting white precipitates were thoroughly washed with excess THF and then dried. The final product was stored in a freezer under 20 ºC.
